# Supplementary material for: Aurones and Flavonols from Coreopsis lanceolata L. Flowers and Their Anti-Oxidant, Pro-Inflammatory Inhibition Effects, and Recovery Effects on Alloxan-Induced Pancreatic Islets in Zebrafish
Source: Molecules. 2021 Oct 9;26(20):6098. doi: 10.3390/molecules26206098 (PMC8540554; doi:10.3390/molecules26206098)
Supplement: Supplementary file 1 [file molecules-26-06098-s001.zip › molecules-1399029-supplementary.pdf]

Supplementary Materials

Aurones and flavonols from *Coreopsis lanceolata* L. flowers and their anti-inflammatory and anti-diabetic effects

Hyoung-Geun Kim [1], Youn Hee Nam [1], Young Sung Jung [2], Seon Min Oh [1], Nguyen Trong Nguyen [1], Min-Ho Lee [4], Dae-Ok Kim [2], Tong Ho Kang [1], Dae Young Lee [3]*, and Nam-In Baek [1]*

[1] Graduate School of Biotechnology and Department of Oriental Medicinal Biotechnology, Kyung Hee University, Yongin 17104, Republic of Korea; zwang05@khu.ac.kr (H.-G.K.); 01030084217@hanmail.net (Y.H.N.); seonmin88@khu.ac.kr (S.M.O.); ntnguyen@khu.ac.kr (N.T.N.); panjae@khu.ac.kr (T.H.K.)

[2] Department of Food Science and Biotechnology, Kyung Hee University, Yongin 17104, Republic of Korea; chembio@khu.ac.kr (Y.S.J.); DOKIM05@khu.ac.kr (D.-O.K.)

[3] Department of Herbal Crop Research, National Institute of Horticultural and Herbal Science, RDA, Eumseong 27709, Korea

[4] Department of Food Technology and Services, Eulji University, Seongnam 461-723, Republic of Korea; agroman@naver.com (M.-H.L.)

* Correspondence: dylee0809@gmail.com (D.Y.L.); nibaek@khu.ac.kr (N.-I.B.); Tel.: +82-43-871-5784 (D.Y.L.); +82-31-201-2661 (N.-I.B.)

Contents:

S1: The isolation procedures of compounds **1–4** from *Coreopsis lanceolata* flowers

S2: ^{1}H and ^{13}C NMR of compound **1**

S3: ^{1}H and ^{13}C NMR of compound **2**

S4: ^{1}H and ^{13}C NMR of compound **3**

S5: ^{1}H and ^{13}C NMR of compound **4**

S6: The materials, equipment, and methods used for quantitative analysis of the isolated flavonoids from *Coreopsis lanceolata* flowers

S7: Cell viability test of extracts, fractions, and compounds **1–4** from *Coreopsis lanceolata* flowers on RAW264.7 cell.

S8: The methods and materials of antioxidant, pro-inflammatory inhibition, and anti-diabetic assay.

S1: The isolation procedures of compounds 1–4 from *Coreopsis lanceolata* flowers

- *Coreopsis lanceolata* flowers (3.1 kg, dry weight (DW)) were extracted in 80% MeOH (67.5 L × 4) at room temperature for 24 h, and then filtered and evaporated to yield a residue of 1.1 kg. The obtained MeOH extracts were suspended in H_2O (2 L) and then successively extracted with EtOAc (2 L × 4) and *n*-BuOH (1.8 L × 4). Each layer was concentrated under reduced pressure to obtain EtOAc (CLFE, 194 g), *n*-BuOH (CLFB, 254 g), and H_2O (CLFW, 652 g) soluble frs.

CLFE (194 g) was put through SiO_2 column chromatography (CC) [13.0 × 17.0 cm, $CHCl_3$–MeOH–H_2O (CMW) 64:6:2→24:6:2→18:6:2→12:8:2, 18 L of all) to afford 13 Frs (CLFE-1 to CLFE-13). CLFE-8 [7.2 g, elution volume/total volume (Ev/Tv) 0.147-0.180] was treated with SiO_2 CC (4.0 × 15.0 cm, CMW (64:6:2, 6.0 L) to provide ten Frs (CLFE-8-1 to CLFE-8-10). CLFE-8-6 (4.7 g, Ev/Tv 0.074-0.087) was put through ODS CC (4.0 × 9.0 cm, MeOH–H_2O (33→ 50→66→75%, 2.0 L of all) to afford nine Frs (CLFE-8-6-1 to CLFE-8-6-9). CLFE-8-6-6 (1.8 g, Ev/Tv 0.200-0.527) was exposed to SiO_2 CC (3.5 × 15.0 cm, CMW (64:6:2, 2.8 L) to produce ten Frs (CLFE-8-6-6-1 to CLFE-8-6-6-10) along with a purified compound **1** [leptosidin, CLFE-8-6-6-7, 139 mg, Ev/Tv 0.580-0.724, SiO_2 TLC R_f 0.64, CMW=28:6:2].

CLFB (254 g) was surrender to SiO_2 CC (13.0 × 15.0 cm, CMW= 72:6:2→50:6:2→30:6:2, 15 L of all) to provide with 14 Frs (CLFB-1 to CLFB-14). CLFB-14 (5.8 g, Ev/Tv 0.317-1.000) was applied to ODS CC (5.0 × 9.0 cm, acetone- H_2O (25%, 5.5 L) to give nine Frs (CLFB-14-1 to CLFB-14-9). CLFB-14-5 (522 mg, Ev/Tv 0.068-0.132) was loaded on ODS CC (5.0 × 8.0 cm, acetone-H_2O (25%, 570 mL) to ultimately give eight Frs (CLFB-14-5-1 to CLFB-14-5-8), compound **3** [isoquercetin, CLFB-14-5-4, 49 mg, Ev/Tv 0.200-0.233, SiO_2 TLC, R_f 0.50, EtOAc-*n*-BuOH-H_2O (EBW)=10:3:1], and compound **2** as well [leptosin, CLFB-14-5-6, 34 mg, Ev/Tv 0.433-0.467, SiO_2 TLC, R_f 0.45, EBW=20:6:2]. CLFB-14-5-8 (40 mg, Ev/Tv 0.500-1.000) was treated with ODS CC (1.5 × 5.0 cm, MeOH-H_2O (23%, 820 mL) to furnish four Frs (CLFB-14-5-8-1 to CLFB-14-5-8-4) and compound **4** as well [astragalin, CLFB-14-5-8-3, 9 mg, Ev/Tv 0.621-1.000, SiO_2 TLC, R_f 0.53, EBW=14:6:2].

S2: ^{1}H and ^{13}C NMR of compound 1

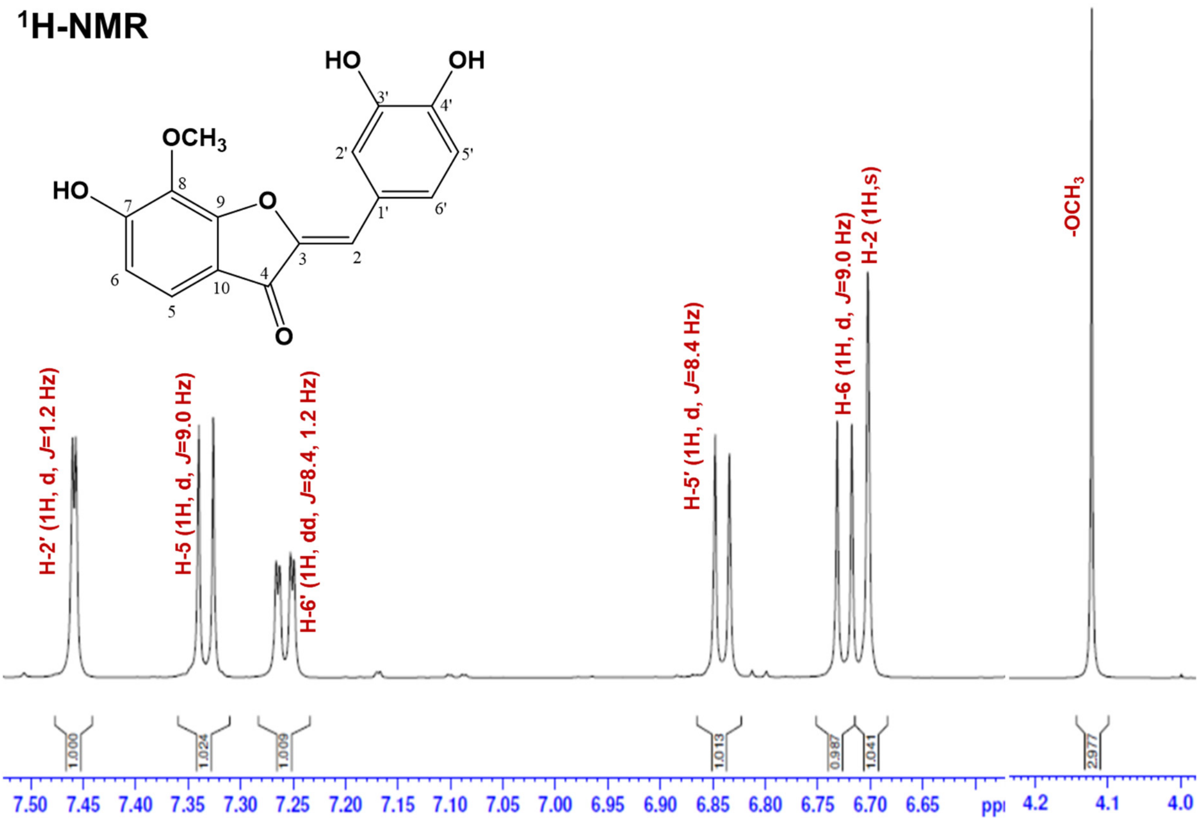

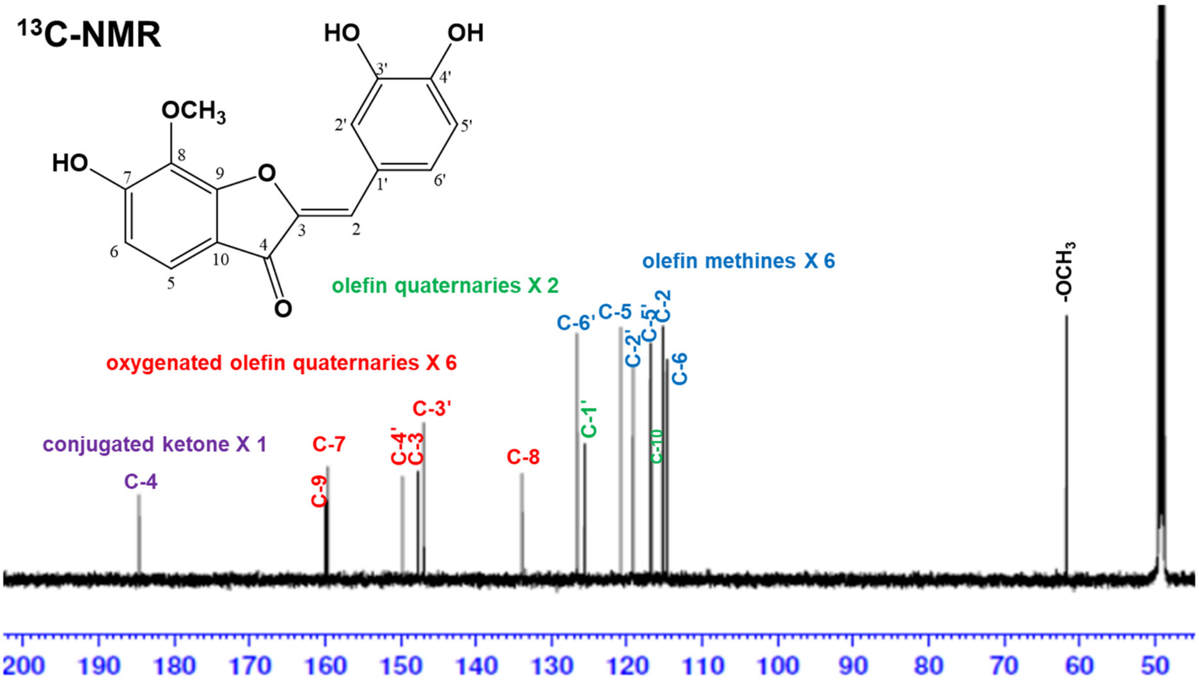

S3: ^{1}H and ^{13}C NMR of compound 2

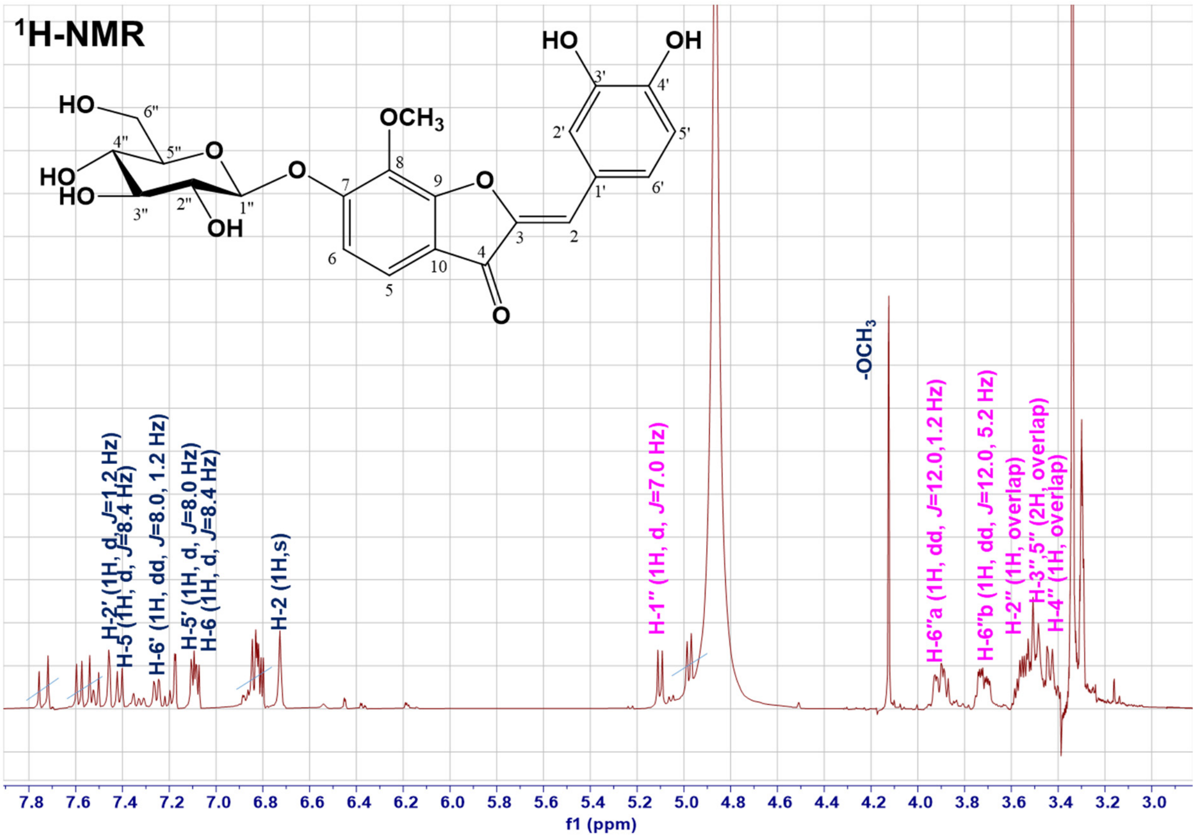

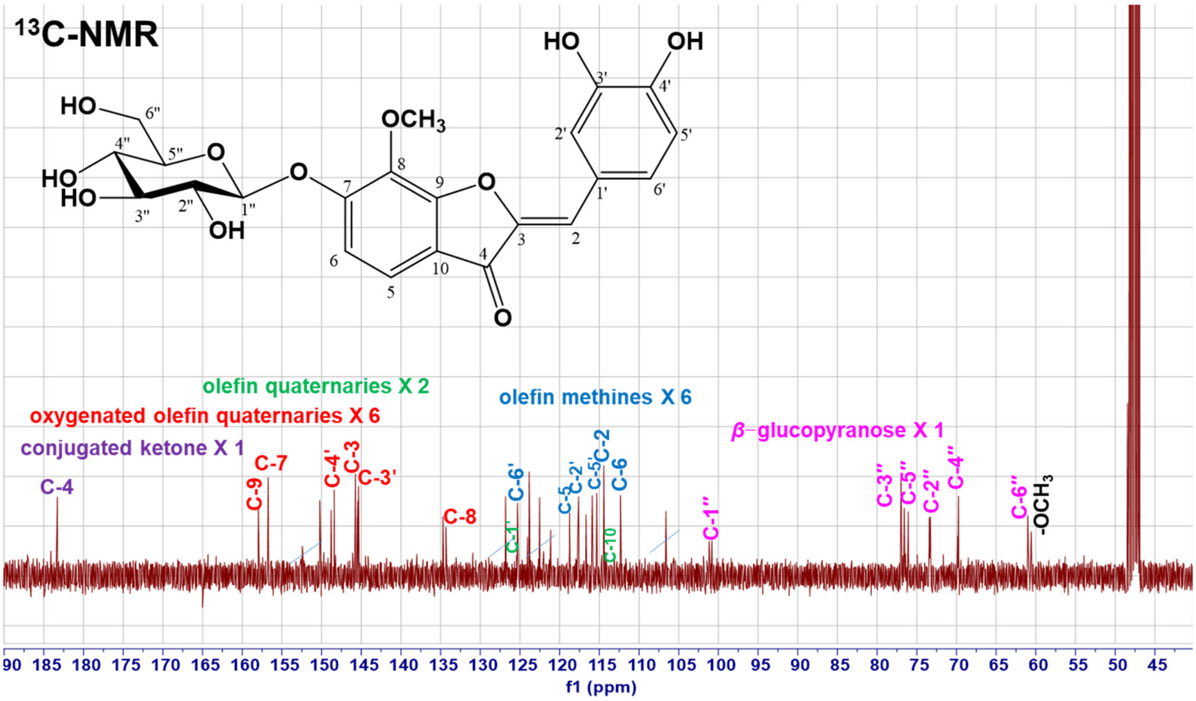

S4: ^{1}H and ^{13}C NMR of compound 3

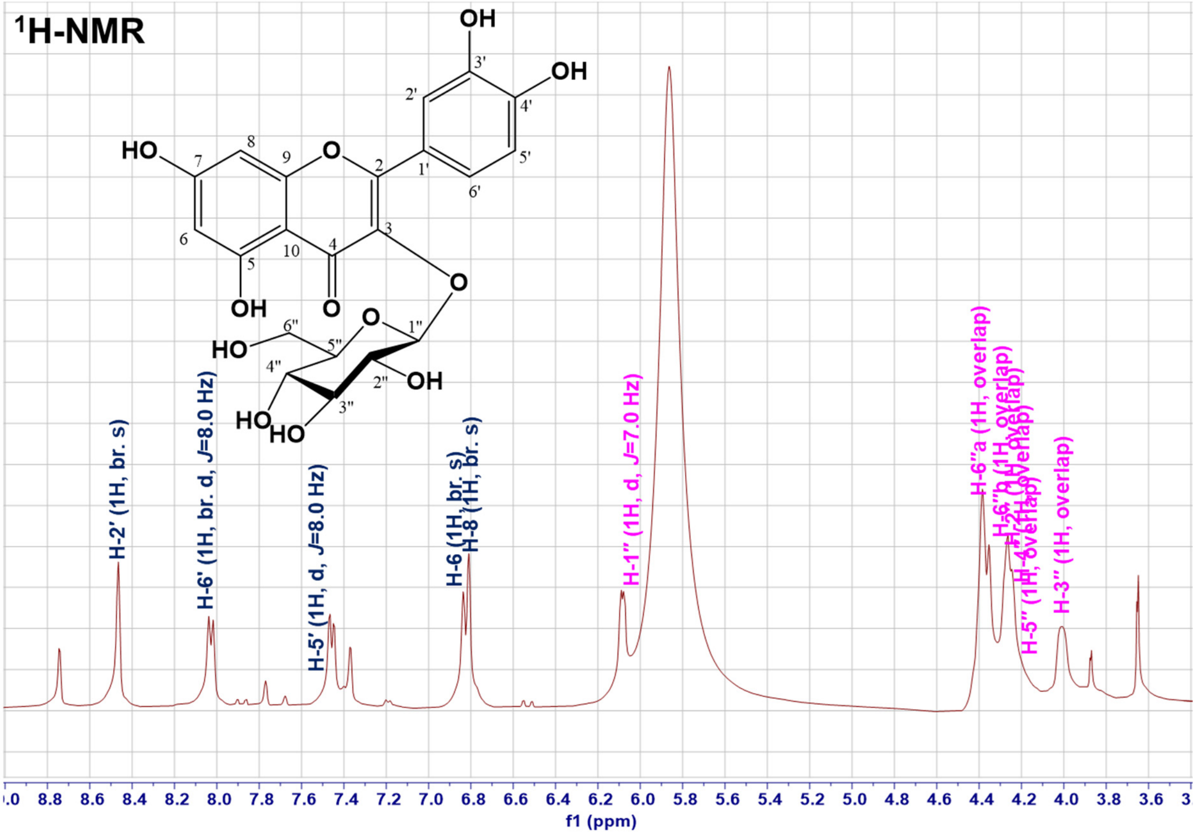

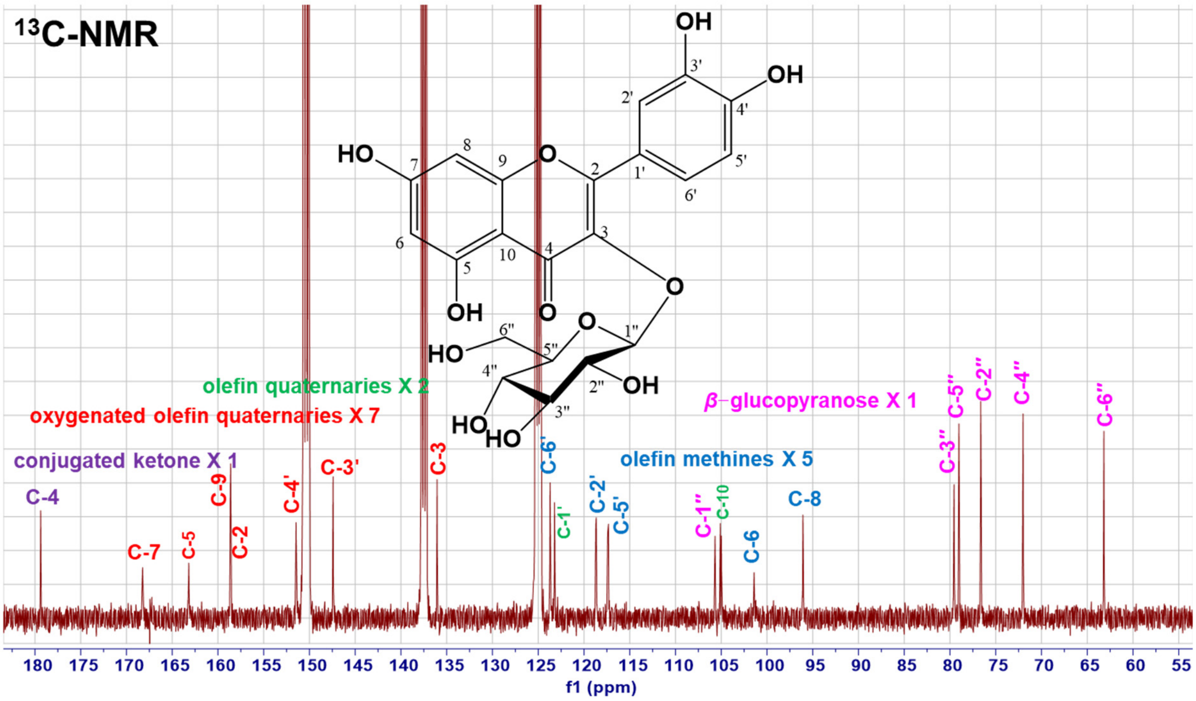

S5: ^{1}H and ^{13}C NMR of compound 4

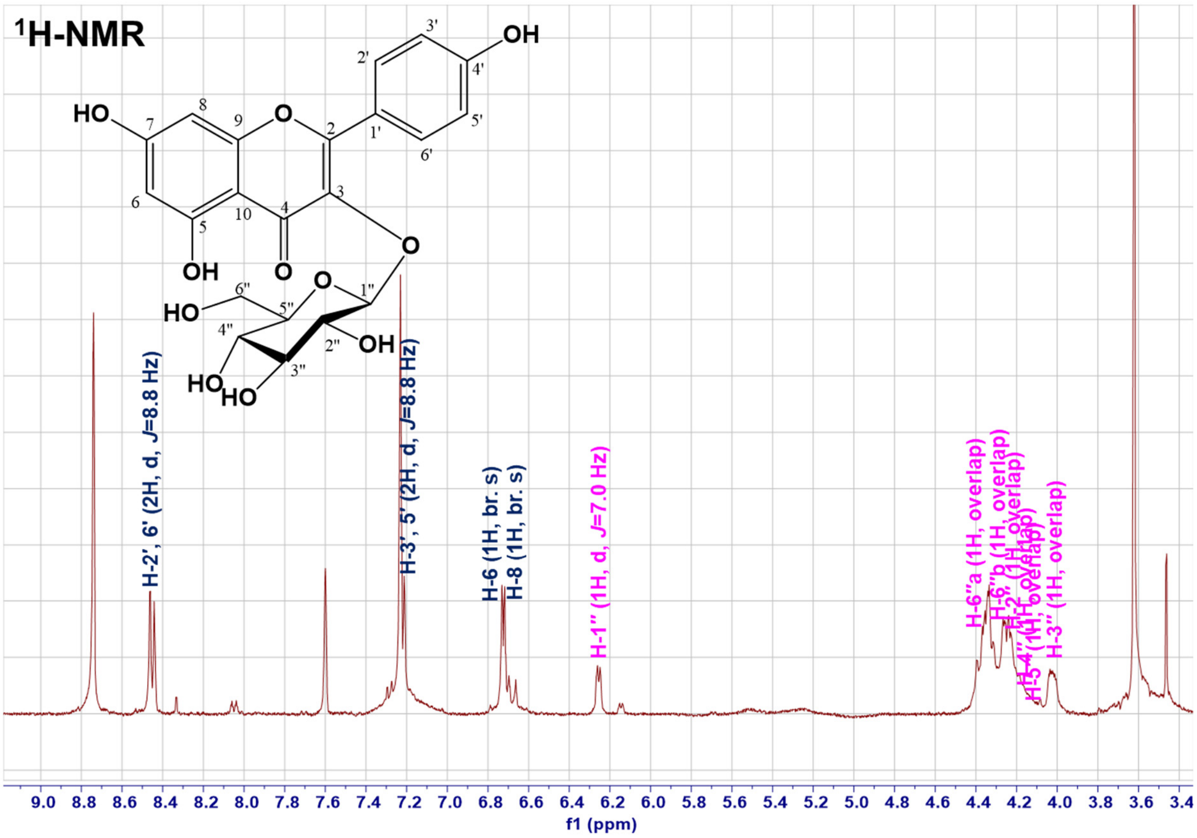

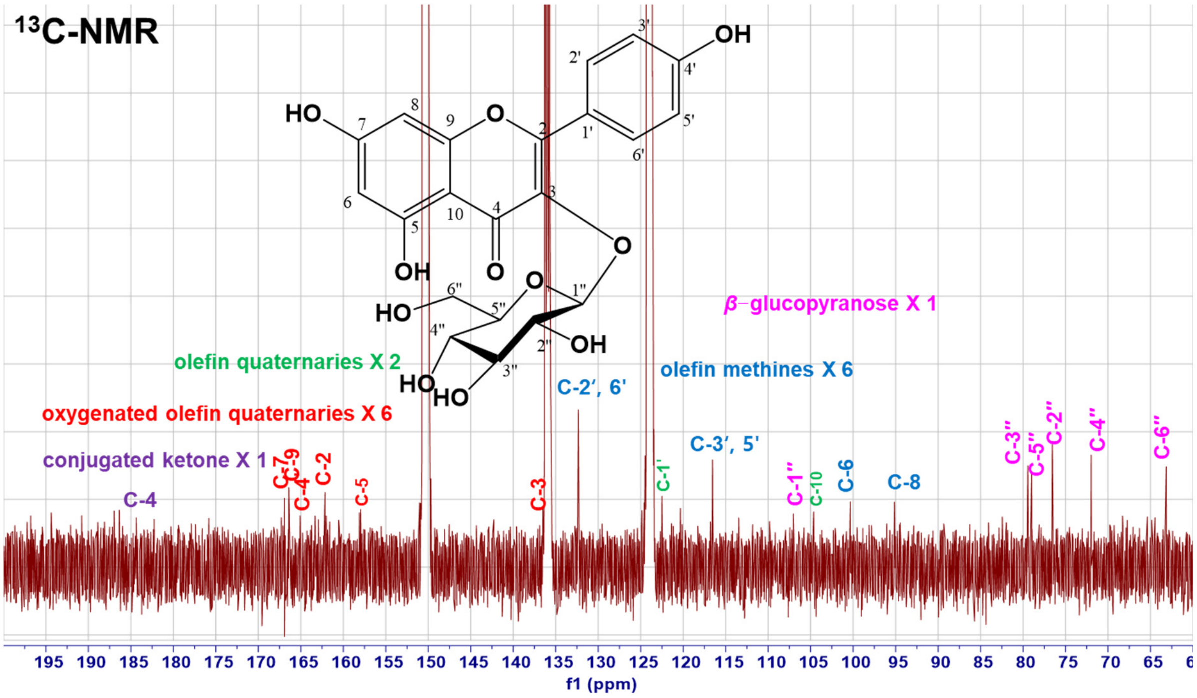

S6: The materials, equipment, and methods used for quantitative analysis of the isolated flavonoids from *Coreopsis lanceolata* flowers

- Quantitative analysis of the isolated flavonoids was performed using HPLC (Shimadzu LC-20; Shimadzu, Kyoto, Japan) with a C18 column (Waters XBridge C_{18}; 120 Å, 5.0 μm, 250 × 4.6 mm; Waters Corp., Milford, MA, USA). The column oven temperature was 40°C, sample injection volumn was 10 μL, and detection wavelength was set to 360 nm. Solvent A (H_2O: formic acid = 99.9: 0.1, v/v) and solvent B (acetonitrile 100%) were used in the mobile phase, and the flow rate was set to 1.0 mL/min. The solvent elution was graded as follows: 5% B at 0 min, 5% B at 3 min, 15% B at 6 min, 20% B at 12 min, 20% B at 35 min, 90% B at 37 min, 5% B at 40 min, and 5% B at 45 min. For the quantitative analysis of flavonoids **1–4** in CLF extract, each flavonoid (1 mg) was precisely weighed and dissolved in 80 % aqueous methanol to make stock solution with a concentration of 1.0 mg/mL. Regression curves were developed for each standard with 5 different concentrations (200, 100, 50, 25, 12.5 μg/mL). CLF (5 mg) was also precisely weighed and dissolved in 80 % aqueous methanol to make stock solution with a concentration of 5.0 mg/mL.

S7: Cell viability test of extracts, fractions, and compounds 1–4 from *Coreopsis lanceolata* flowers on RAW264.7 cell.

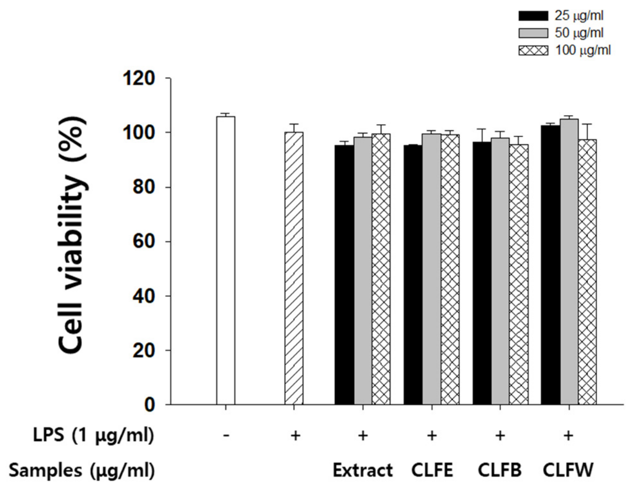

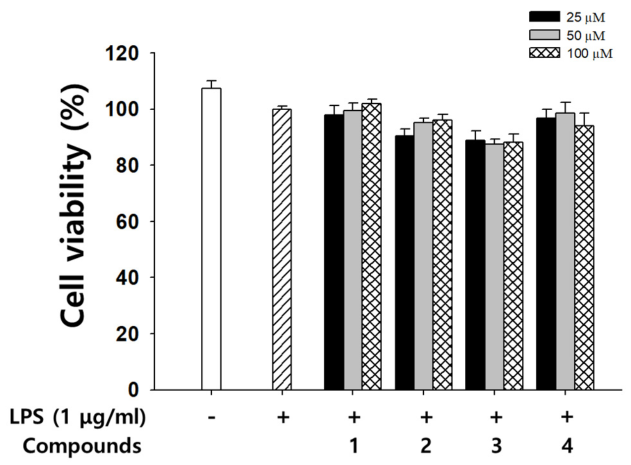

S8: The methods and materials of antioxidant, pro-inflammatory inhibition, and anti-diabetic assay.

1. Antioxidant activities

1.1. Free Radical Scavenging Activity

Antioxidant capacities were determined using the 2,2′-azino-bis(3-ethylbenzothiazoline-6-sulfonic acid) (ABTS) and 2,2-diphenyl-1-picrylhydrazyl (DPPH) radical scavenging assays. Briefly, the ABTS radical solution was adjusted to an absorbance of 0.650±0.020 at 734 nm. Reactions between the ABTS radical solution and the diluted extract, fractions, and compound 1–4 (satisfying the standard curve range) were allowed to proceed at 37°C for 10 min, and decreases in the absorbance of the resulting solution were measured using a spectrophotometer (SPECTRONIC 200; Thermo Fisher Scientific Inc., Waltham, MA, USA). For the DPPH assay, the absorbance of DPPH radicals in 80% (v/v) aqueous methanol was set to 0.650±0.020 at 517 nm. Reactions between the DPPH radical solution and the diluted extract, fractions, and compound 1–4 (satisfying the standard curve range) were allowed to proceed at ambient temperature for 30 min. Decreases in the absorbance of the resulting solution were monitored at 517 nm using a spectrophotometer (SPECTRONIC 200). Antioxidant capacities were expressed as mg vitamin C equivalent (VCE)/100 mg.

1.2. Cell Culture and Cytotoxicity Assessment

For the cell culture, a complete medium (with the addition of 10% heat-inactivated fetal bovine serum (FBS), 100 units/mL penicillin, and 100 μg/mL of streptomycin) was used. RAW 264.7, HepG2, and Caco-2 cells were cultured in complete Dulbecco's modified Eagle's medium (DMEM), and PC-12 cells were cultured in complete Roswell Park Memorial Institute (RPMI) 1640 medium. All cell lines were sub-cultured when approximately 90% of the distribution was in the culture dish. To determine the non-toxic maximal concentration of compound 1–4, cytotoxicity was assessed using a 3-(4,5-dimethylthiazol-2-yl)-2,5-diphenyl-tetrazolium bromide (MTT) reduction assay. PC-12, Caco-2, HepG2, and RAW 264.7 cells were seeded at a density of 2 x 10^4 cells/well in a 96-well plate in RPMI 1640 or DMEM medium containing FBS and left for 24 h. After removing the medium, cells were treated with a serum-free medium containing compound 1–4 at various concentrations. Following a 24 h incubation period, the medium was removed from each well. Then, MTT reagent was added, the plate was incubated for 3 h, and 50 μL of DMSO was added. The absorbance was measured using a microplate reader (Infinite M200; Tecan Austria GmbH, Grödig, Austria) at 570 nm (test wavelength) and 630 nm (reference wavelength). The cytotoxic effect was expressed as the percentage (%) of metabolically active cells relative to control cells cultured without test samples.

1.3. Measurement of Intracellular Oxidative Stress

Intracellular OS levels were evaluated using 2′,7′-dichlorofluorescein diacetate (DCFH-DA) following previously reported methods [manuscript ref 43]. Briefly, Caco-2, HepG2, RAW 264.7 and PC-12 cells were seeded (2 x 10^4 cells/well in a 96-well plate) in their respective complete media and incubated for 3h in a humidified incubator with 5% CO_2 at 37°C. The cells were then treated with non-toxic concentrations (0.625–10 μg/mL) of compound 1–4. After removing the supernatant, 50 μM DCFH-DA in phosphate-buffered saline (PBS) was added and incubated for 30 min, and then the cells were treated with 200 μM H_2O_2 for 30 min. Fluorescence was measured using a microplate reader (Infinite M200) with excitation at 485 nm and emission at 530 nm.

2. Pro-inflammatory inhibiton activity

2.1. Determination of NO Production

NO produced by RAW 264.7 cells was determined using a method reported in the literature [32]. In brief, RAW 264.7 cells at a density of 2 × 10^4 cells/well in a 96-well plate were pre-cultured for 24 hr. The cells were then stimulated with 1 μg/mL LPS in the pres-ence of samples for 24 hr. Supernatant was obtained to evaluate the nitrite level using the Griess reagent system. The nitrite level was determined by measuring the absorbance at 540 nm with a microplate reader (Infinite M200, Tecan, Männedorf, canton of Zürich, Switzerland).

2.2. Assays for IL-1β, IL-6, and TNF-α

The culture medium was collected to determine the levels of IL-β, IL-6, and TNF-α present in each sample using ELISA kits tailored to each collection process (R&D Systems,Inc.), as per the manufacturer's instructions. Briefly, RAW264.7 cells were seededin 48-well culture plates at a density of 5 x 10^5 cells/well. After incubation, the supernatant was collected and used in the cytokine ELISA kits for measuring the concentrations of IL-β, IL-6, and TNF-α.

2.3. Western Blot Analysis for Protein Expression

The intracellular content of pro-inflammatory enzymes was measured by Western blotting. RAW 264.7 cells at a density of 2 x 10^6 cells/well were pre-cultured in a 6-well plate for 24 h and then stimulated with 1 μg/mL LPS and 1 ng/mL *β*-actin in the presence of samples for 24 h. Total cell extracts were obtained by a lysis buffer (50 mM Tris-HCl, pH 7.5; 150 mM NaCl; 1mM EDTA; 20 mM NaF; 0.5% NP-40; 1% Triton X-100) containing a protease inhibitor cocktail (GenDEPOT, Barker, TX,USA). The protein concentration was quantified using the Bradford assay. Cell extracts were loaded onto an 12% sodium dodecyl sulfate polyacrylamide gel and transferred to nitrocellulose membranes. The membranes were incubated with iNOS (1:500) and COX-2 (1:500) overnight at 4°C. Subsequently, horseradish-peroxidase-conjugated anti-mouse or anti-rabbit secondary antibody was used for 1 h at ambient temperature. Blots were detected using EzWestLumi plus (ATTO, Tokyo, Japan) and analyzed using an EZ-Capture MG (ATTO). The band density was quantified using Image J software (Bogdan, 2001, Stuttgart, Baden-Wurttemberg, Germany).

3. Antidiabetic activity

3.1. Chemicals

Alloxan and sea salts were purchased from Sigma-AldrichCo. (St. Louis, MO, USA). 2-(N-(7-nitrobenz-2-oxa-1,3-diazol-4-yl)amino)-2-deoxyglucose (2-NBDG) was purchased from Thermo Fisher Scientific Inc. (Ganseville, FL, USA). Diazoxide was purchased from Santa Cruz Biotechnology Inc. (Paso Robles, CA, USA). Glimepiride was purchased from Cayman Chemical Co. Inc. (Arbor, MI, USA).

3.2. Animals

Adult zebrafishes were maintained in a zebrafish S type system (1500[W] × 400[D] × 2050 [H] mm) (Daejeon, Korea) and a 14-h light:10-h dark cycle at 28.5°C. Two pairs of adult zebrafishes were placed in a spawning box overnight to obtain zebrafish larvae. The next day, the zebrafish spawned during a 30-min period of light. Zebrafish embryos were then collected at 3 hours post-fertilization for incubation and maintained in 0.03% sea salt solution in a 14:10 h light-dark photocycle in an incubator at 28.5°C.

3.3. Ethics Statement

All zebrafish experimental procedures were carried out in accordance with standard zebrafish protocols and were approved by the Animal Care and Use Committee of Kyung Hee University [KHUASP(SE)-15-10].

3.4. Evaluation of Recovery Efficacy on Pancreatic Islet damaged by Alloxan in Zebrafish

Zebrafish larvae (n=10) were divided into the following groups: normal group, alloxan-induced group (control), and alloxan-induced groups treated with samples. Wild-type zebrafish larvae 6 days post-fertilization (dpf) were placed into 24-well plates. The larvae were exposed to 600 μM alloxan for 3 hours to induce pancreatic islet damage. To determine the efficacy of samples, the alloxan-induced larvae were treated with 1 μg/mL extracts for 3 h, then stained for 30 min with 40 μM 2-NBDG and rinsed with 0.03% sea salt solution for 20 min. After staining, pancreatic islets were observed under a fluorescence microscope (Olympus 1X70 microscope; Olympus Co., Tokyo, Japan) and analyzed using Focus Lite software (Focus Co, Daejeon, Korea) was used for image analysis.

3.5. Action of diazoxide on alloxan-induced diabetic zebrafish

Five dpf wild-type zebrafish were placed into 24-well plates. Zebrafish larvae were divided into the following twelve groups: normal, normal treated with diazoxide or alloxan, and alloxan-induced diabetic zebrafish treated with diazoxide, Glimepiride, Glimepiride + diazoxide, CLFW, CLFW + diazoxide, compound 1 (F1), F1 + diazoxide, compound 2 (F2), F2 + diazoxide, compound 3 (F3), F3 + diazoxide, compound 4 (F4), F4 + diazoxide. Compounds and extracts were applied at 1 μg/mL and diazoxide at 25 μM. The zebrafish larvae were treated with 600 μM concentrations of alloxan for 3 h and then the solution was changed to 0.03% sea salt solution. After 3 h, alloxan-induced zebrafish larvae were treated (or co-treated) with respective compounds or extracts for 3 h. Following treatment, zebrafish larvae were stained with 40 μM 2-NBDG for 30 min and rinsed with 0.03% sea salt solution for 20 min to obtain pancreatic islet images. After staining, pancreatic islet images were visualized using fluorescence microscopy and analyzed using Focus Lite.

4. Statistical Anlaysis

Results (mean ± SEM, n=3) were assessed using one-way analysis of variance and the Tukey–Kramer honestly significant difference (HSD) test with $p < 0.05$ considered to represent statistical significance. All statistical analyses were performed using SPSS 22.0 (SPSS Inc., Chicago, IL, USA).
